# Preeclampsia Management and Maternal Ophthalmic Artery Doppler Measurements between 19 and 23 Weeks of Gestation

**DOI:** 10.3390/jcm13040950

**Published:** 2024-02-07

**Authors:** Elitsa Gyokova, Eleonora Hristova-Atanasova, Georgi Iskrov

**Affiliations:** 1 Department of Obstetrics and Gynecology, Faculty of Medicine, Medical University-Pleven, 5800 Pleven, Bulgaria; elitca.gaokova@mu-pleven.bg; 2 Obstetrics Clinic, UMHAT “Saint Marina” Pleven, 5800 Pleven, Bulgaria; 3 Department of Social Medicine and Public Health, Faculty of Public Health, Medical University of Plovdiv, 4000 Plovdiv, Bulgaria; georgi.iskrov@mu-plovdiv.bg

**Keywords:** preeclampsia, ophthalmic artery, PSV2/PSV1, management, Bulgaria

## Abstract

**Background**: The ophthalmic Doppler is a reliable and impartial way to assess the severity of preeclampsia (PE). The study aimed to assess the potential utility of Doppler measurements of the maternal ophthalmic arteries during the weeks 19–23 of gestation, both independently and in combination with established biomarkers for PE. **Methods:** A prospective cohort study was conducted involving women who were recruited from a variety of standard appointments, including booking, scanning, and regular prenatal visits. A total of 200 women that were divided into high-risk and low-risk groups for developing PE were involved during the period between April 2023 and November 2023. **Results:** The ophthalmic ratio had significantly higher values in high-risk patients than in low-risk women (*p* = 0.000). There was a significant relationship between PSV2/PSV1 and gestational age at birth in women with PE compared to the ones who did not develop PE. **Conclusions:** An ophthalmic artery Doppler can play a crucial role in the early detection of PE, allowing for timely intervention and management. Incorporating the ophthalmic artery Doppler as a screening tool for PE in Bulgaria has the potential to improve early detection, risk stratification, and overall maternal and fetal health outcomes.

## 1. Introduction

Over the last decades, different definitions of preeclampsia (PE) have been suggested. According to the International Society for the Study of Hypertension in Pregnancy (ISSHP), a woman is considered to have PE if she develops high blood pressure (systolic blood pressure (SBP) of ≥140 mmHg and/or diastolic blood pressure (DBP) of ≥90 mmHg) for the first time at least 20 weeks into her pregnancy and has proteinuria (≥300 mg/24 h or a protein-to-creatinine ratio >30 mg/mmol or ≥2+ on dipstick testing) [1]. Later, the ISSHP definition of PE was reviewed, including cases without proteinuria but with evidence of hematological, renal, or hepatic impairment [2]. As one of the most serious maternal complications during pregnancy, the accurate prediction of PE onset and progression, as well as the prevention, are the three fundamental elements that should be improved, so the associated PE morbidity and mortality can be effectively lowered [3,4,5].

The ophthalmic artery serves as a practical pathway for Doppler assessment that offers insight into the less approachable cerebral circulation [6]. It shares morphological and functional similarities with the intracranial vasculature [7,8]. Approximately 30% to 100% of women diagnosed with PE may experience ocular complications [9,10,11]. The ocular Doppler is a reliable and impartial way to assess the severity of PE. It is a very easily accessible vessel to measure and a promising marker in screening for PE [7,12,13,14,15,16].

According to a study by Sapantzoglou’s group, either used alone or in conjunction with other biomarkers, the ophthalmic artery peak of systolic velocity (PSV) ratio between 19 and 23 weeks of pregnancy may help predict the development of PE later on, especially preterm PE [17]. If taken as an individual parameter, it has better performance in predicting PE compared to mean arterial pressure (MAP), uterine artery pulsatility index (UtA-PI), placental growth factor (PlGF), or soluble fms-like tyrosine kinase-1 (sFlt-1). The only ophthalmic artery index that reliably predicted PE was the ratio of the second to the first PSV ratio. Another study by Sarno et al. showed that the PSV ratio, when used alone or in conjunction with other biomarkers, accurately predicted the future occurrence of PE [18]. According to Selima et al., ophthalmic artery peak ratio (PR) values of less than 0.72 can suggest PE with exceptional specificity of 81.3% and sensitivity of 90.5% [19]. However, despite the high predictive value, the PSV ratio remains a predictive modality rather than a diagnostic tool. Currently, there are no clear-cut recommendations from the Bulgarian Obstetrics and Gynecology Society to routinely screen for PE in the first trimester [20]. Nevertheless, it does recommend a low dose of aspirin (150 mg) started before 16 weeks of gestation for those patients with one of the following risk factors: previous PE, chronic hypertension, chronic kidney disease, diabetes type I and II, autoimmune diseases, such as systemic lupus erythematous, or antiphospholipid syndrome [18]. A sizable number of patients in Bulgaria seek care from the private medical sector, where they undergo standard screenings for PE during the first trimester using maternal history, serum biomarkers (PAPP-A, PlGF), MAP, and UtA-PI. Unfortunately, there are no standards or regulations for the care provided in the private medical sector, and nor are there data for the results. As a result, the patients’ first appointments with fetal medicine specialists are typically during weeks 19 to 23 of gestation for the anomaly scan. The ophthalmic artery scan has not been carried out in the country so far, except for research purposes. The study aimed to assess the potential utility of Doppler measurements of the maternal ophthalmic arteries during weeks 19–23 weeks of gestation, both independently and in combination with established biomarkers for PE.

## 2. Materials and Methods

### 2.1. Study Design and Participants

A prospective cohort study was conducted involving women who were recruited from a variety of standard appointments, including booking, scanning, and regular prenatal visits. We applied the purposive sampling technique to select potential participants. 

We used a combination of two inclusion criteria: a singleton pregnancy that was evaluated between 19 + 0 and 22 + 6 weeks of gestation, and a maternal age between 18 and 46 years. The exclusion criteria were as follows: fetal abnormalities, aneuploidy, chronic hypertension, pre-gestational diabetes, connective tissue disorders, pre-existing eye problems, congenital heart diseases in either the mother or the fetus, and maternal infections including HIV, HBV, or HCV at the time of screening. Written informed consent was obtained for all cases.

This study was funded by the “Young Scientists and Postdoctoral Students 2” National Programme of the Bulgarian Ministry of Education and Science. The research was performed by the Department of Obstetrics and Gynecology at the Medical University—Pleven and University Hospital “Saint Marina” in Pleven, Bulgaria. Data collection took place from April 2023 to November 2023. A total of 217 women volunteered to take part in the survey, 17 of whom later dropped out for personal reasons or lack of information. Before taking any measures, the obstetrician for the study evaluated each woman and certified the well-being of the mother and the fetus. Every non-invasive test and ultrasound performed throughout the cohort followed the same set of rules, took place in the same room, and used the same operator.

During their appointment at 19–23 weeks, the following procedures were performed: (1) Collection of demographic information and medical history of the women; (2) evaluation of the fetus’ growth and anatomy; (3) the blood pressure was measured, and the MAP was calculated by the following formula: MAP = DP + 1/3 (SP − DP) or MAP = DP + 1/3 (PP); (4) color flow imaging was employed to determine the mean UtA-PI transabdominally; (5) a blood sample was taken for the concentrations of PlGF and sFlt-1 in the serum to be assessed by a standardized laboratory; (6) the competing risks model was used to figure out how likely it was that a specific patient would develop PE at any given time. This model combined biomarkers with the medical history and demographic information of the mother; (7) the Fetal Medicine Foundation (FMF) Calculator was used to figure out the risk of developing PE between 19 and 23 weeks by a combination of maternal characteristics and medical history, including UtA-PI, MAP, sFlt-1, and PlGF [13,14]. The final sample of 200 participants was divided into two groups: high-risk patients (*n* = 60) and low-risk patients (*n* = 140). High risk was defined as a risk of more than 1 in 150 for developing PE before the 37th week. Low risk was defined as <1 in 150 for developing PE before 37 weeks of gestation [15,16]; (8) after that, an examination was conducted on the flow velocity wave morphologies of the maternal ophthalmic arteries of both eyes. We used the FMF method to predict PE in our study, along with a new way of predicting by measuring the ophthalmic artery PSV2/PSV1 ratio.

All patients agreed to be contacted after the delivery to report the outcome of the pregnancy and, specifically, the incidence of PE if necessary. We collected data about the delivery and the complications throughout the pregnancy. 

### 2.2. Doppler Measurements of the Ophthalmic Arteries 

**Figure 1:**
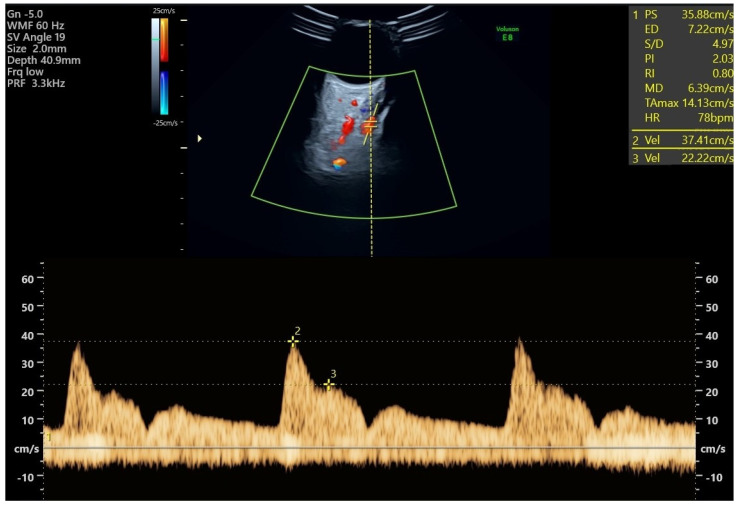
Ophthalmic artery Doppler study: an ultrasound image shows the ophthalmic artery’s color flow. The ocular artery flow velocity waveform, which shows the first and second systolic velocities, is displayed at the bottom and was obtained using pulsed-wave Doppler imaging.

All study participants had Doppler measurements of both ophthalmic arteries taken at their regular visit at 19 + 0 to 22 + 6 weeks of gestation. PE screening utilizes three indices: the ratio of the second peak systolic velocity to the first (PSV2/PSV1), the first peak systolic velocity (PSV1), and the second peak systolic velocity (PSV2) [17,18,19]. The expectant individual ought to be positioned supine and allowed to undergo a five-minute period of rest prior to undergoing the measurements. It is recommended to have the eyes closed. Following the application of lubricant, a transversely oriented linear transducer operating at 7.5 MHz is positioned on the eyelid. The ophthalmic artery ought to be readily distinguishable via color Doppler. The vessel is situated in a medial and superior position along the optic nerve, manifesting as a hypoechogenic band on the scan [20]. The test needs to be carried out using a pulsed-wave Doppler instrument with a 2 mm sample gate, a 125 kHz pulse repetition frequency, a 50 Hz high-pass filter, and an angle of insonation less than 20 degrees. Three to four vibrations ought to be recorded (Figure 1). To prevent untoward effects, the duration of the examination for each eye should not exceed a few seconds. To optimize performance, it is recommended to acquire two measurements for each eye [21,22,23,24]. The operator had previously passed practical assessment in obtaining and analyzing Doppler measurements of the ophthalmic artery and conducted measurements for more than 12 months before enrolling patients in the study.

### 2.3. Outcome Measures 

The criteria for assessing PE outcomes were based on the ISSHP guidelines. These were systolic blood pressure > 140 mm Hg or diastolic >90 mm Hg at least twice, 4 h apart, emerging after 20 weeks of gestation in previously normotensive women, and at least one of the following: (1) renal insufficiency with serum creatinine > 97 µmol/L without any other kidney disease; (2) hepatic dysfunction with higher blood transaminase levels; (3) proteinuria (≥300 mg/24 h or protein to creatinine ratio > 30 mg/mmoL or >2 + on dipstick testing).

We classified the participants into two groups depending on the pregnancy outcome: group one, patients who developed PE, and group two, patients who did not develop PE. The severity of PE in group one was not evaluated because there was only one patient in this group who had features of severe PE (HELLP syndrome).

### 2.4. Ethical Considerations

The study was approved by the Ethics Committee of the Medical University—Pleven (IRB number 723/31 March 2023) and was conducted in accordance with the Declaration of Helsinki and the guidelines of Good Clinical Practice. Written informed consent was obtained for all cases. All records were anonymized and de-identified prior to the analyses.

### 2.5. Data Analysis

Data were analyzed using SPSS (version 26.0; SPSS, Inc., Chicago, IL, USA). Figures and charts were generated using R version 4.3.1, the ggplot2 package version 3.3.2, and Microsoft 365 Office. All numeric variables were tested for normal distribution by applying the Kolmogorov–Smirnov test. The continuous data were presented as the mean ± standard deviation of the mean (SD). Numbers and percentages (*n*, %) were used to report qualitative variables. The chi-square test was applied to assess the association between two categorical variables. The Pearson’s correlation coefficient test was used to evaluate the correlations between the PSV2/PSV1 ratio and the PE biomarkers. For statistical analysis, the one-way analysis of variance (ANOVA) and a Bonferroni post hoc test were utilized. For all tests, a level of significance of *p* < 0.05 was adopted.

## 3. Results

A total of 200 women with a mean age of 34.1 ± 5.0 years (range, 18–48 years) and a mean gestational age of 21.1 ± 0.6 weeks took part in the study, 88.5% (*n* = 177) of whom were nulligravida. The majority had spontaneous methods of conception; only 12% (*n* = 24) smoked, and 6.5% (*n* = 13) had gestational diabetes mellitus (GDM). The statistically significant differences between the high-risk and low-risk groups were in terms of the mean age and the conception method (*p* < 0.05). There was no significant difference in the BMI between the two groups; more than 70% had a BMI of ≤30 kg/m^3^ (*p* > 0.05). In the group of women who were considered to be at high risk, the average age was 35.4 ± 4.9 years, more than 16.7% (*n* = 10) of them had conceived by in vitro fertilization (IVF), and the average gestation week of delivery was 38.6 ± 2.5. As expected, the PSV2/PSV1 ratio, mean UtA-PI, UtA-PI MoM, MAP, MAP MoM, and sFLT-1/PlGF ratio were all greater in the high-risk group (*p* < 0.05).

During pregnancy, 20 women developed PE, corresponding to an incidence rate of 10%. The average time between the Doppler examination (inclusion of patients in the study) and the development of PE was 16.6 ± 2.8 weeks. The disease occurred at an average of 36.2 ± 2.8 weeks’ gestation. The percentage of participants who had a prior PE was 10% (*n* = 20), and only two of those women acquired a PE (Table 1). Table 1 presents the demographic characteristics and risk factor exposure of the study population.

Every patient who developed PE had an SBP of at least 140 mmHg (range: 140–180 mmHg) and a DBP of at least 87 mmHg (range: 87–100 mmHg). Proteinuria was observed in 55% (*n* = 11) of women diagnosed with PE. It was diagnosed according to one of the subsequent criteria: (1) 300 mg/24 h for at least five patients; (2) 30 mg/mmoL protein to creatinine ratio for at least four patients; (3) 2+ dipstick testing for at least two patients. The remaining nine women who did not develop PE had indications of hematological, renal, or hepatic impairment but no protein in their urine.

Before their 16 weeks of GA, 9% (*n* = 18) of all observed patients at risk of developing PE were administered 150 mg of acetylsalicylic acid (ASA) once daily. It was determined that 11 of them were at high risk (Table 1), but only 5 of 18 (27.7%) developed PE. ASA was given to a mere four patients due to their prior medical history of PE or FGR. A mere six patients were prescribed ASA on account of prior assisted reproductive procedures, while eight patients were prescribed ASA due to their high body mass index or advanced age (>40 years).

No prescription for ASA was issued to the remaining patients who were recruited. As these subjects were not enrolled in the research until their 19th week of gestation, the study team did not prescribe it due to the increased risk of placental abruption. Notably, the number of patients included in the study is negligible in comparison to the amount of ASA consumed, which limits the exponential data available in the present study.

**Figure 2:**
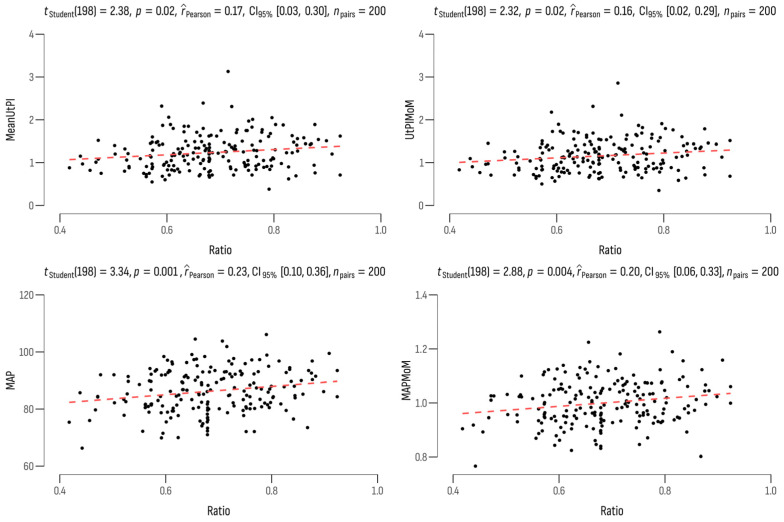
Correlations between the PSV2/PSV1 ratio and the PE biomarkers.

We found a weak positive correlation between the PSV2/PSV1 ratio on the one hand and the mean UtA-PI (r = 0.17; *p* = 0.018), the UtA-PI MoM (r = 0.16; *p* = 0.021), the MAP (r = 0.23; *p* = 0.001), and the MAP MoM (r = 0.20; *p* = 0.004) on the other (Figure 2). A significant increase in the ophthalmic ratio indicator was observed in patients who were over the age of 35 years (0.71 ± 0.11; *p* = 0.004) and who had a BMI greater than 30 kg/m^3^ (0.70 ± 0.11; *p* = 0.050). 

Significantly, deliveries occurred at 37.9 ± 2.3 gestational years (*p* = 0.003) in the majority of women who developed PE, and 90% (*n* = 18) of them were nulligravida. The effectiveness of PE screening was evaluated in cases with and without a PE, and the results indicated that the ophthalmic ratio, MAP, and MAP MoM were significantly higher in the group of patients who developed a PE (*p* < 0.05) (Table 2).

## 4. Discussion

The objective of this research was to evaluate the reliability of Doppler measures of ophthalmic arteries between weeks 19 and 23 of pregnancy in identifying PE as a single method and along with validated indicators of PE. The demographic characteristics of high-risk and low-risk groups differed significantly in this study, with regard to maternal age and method of conception. These findings suggest that the specific method by which a woman conceives has a substantial influence on her individual risk of developing PE. According to the present data, the mean UtA-PI and UtA-PI MoM values were considerably greater in high-risk patients compared to low-risk women (*p* < 0.05). Using the PSV ratio, our research demonstrated that an ophthalmic artery Doppler could enhance the efficacy of PE screening and performed better in PE prediction than UtA-PI, PlGF, and sFlt-1.

In general, the findings indicated that maternal ophthalmic artery Doppler could serve as a valuable tool in identifying pregnant women who have an increased susceptibility to developing PE during the course of the pregnancy through routine screening. This finding is also in correlation with previous studies with the same outcome [5,6,7,8,12,13,14,15,16,17,25,26].

The research’s findings indicate that the mean age of participants in the high-risk PE group was 35.4 ± 4.9 years, while it was 33.6 ± 4.9 years in the low-risk group. According to Moreno et al., similarly, the mean maternal age in both groups was 33 years old, but the group that developed PE had twice as many women over 35 and 40 years old as the control group [27]. Based on our results, it appears that pregnancies that happen naturally seem to be less likely to be affected by PE than pregnancies that happen with the help of assisted reproductive technologies, especially IVF. This intriguing observation aligns with the conclusions drawn by Thomopoulos et al., reinforcing the notion that the method of conception may be a crucial determinant in the occurrence of PE [28].

In response to our outcomes, the MAP and MAP MoM of the high-risk group of women were significantly greater at the time of scanning than those of the low-risk group. The MAP showed discernible variations between the groups who developed PE and those who did not. This was previously confirmed when Wright et al. created a model to express the MAP in MoM to avoid the MAP being affected by maternal characteristics and medical history, such as maternal age, gestational age, racial origin (Afro-Caribbean), weight, smoking, previous history of PE, family history of PE, chronic hypertension, and diabetes mellitus [29]. Subsequent research confirmed that in pregnancies that develop PE, MAP is increased, and the separation in MoM values from normal is greater earlier than later in gestation age. The performance of screening based on maternal characteristics, medical history, and MAP improves with advancing gestational age during the screening period [30,31].

Papageorghiou et al. performed a multicenter screening for PE and fetal growth restriction, which showed that in the second trimester, the mean UtA-PI above the 95th centile had a sensitivity of 41% for predicting PE development [32]. In another study by the group of Lees, it was also confirmed that the mean UtA-PI at 23 weeks above the 95th centile was proven to be associated with poor pregnancy outcomes, such as fetal growth restriction, PE, placental abruption, and intrauterine demise [33]. A recent study advocates that decreased PI of the uterine artery is linked to the onset of PE and suggests that poor placentation is the foundation of the pathogenesis [34]. Moreover, our findings provided complete support for this hypothesis.

There was a significant relationship between PSV2/PSV1 and gestational age at birth in women with PE compared to the ones who did not develop PE. The result shows that as the PSV2/PSV1 ratio increases, the gestational age at birth decreases, and vice versa. These facts demonstrated that Doppler measurements of the maternal ophthalmic arteries in conjunction with established biomarkers for PE have the potential to be useful. Previous studies also supported those findings. Matias et al. carried out a pilot study with 347 pregnant women between weeks 20 and 28. They found differences in ophthalmic artery Doppler measurements between women who were preeclamptic and women who were not affected by the condition: the detection rate (DR) was 70% and the false positive rate (FPR) was 25% [35]. A large observational study by Sapantzoglou et al. was carried out between 19 and 23 weeks and showed that the ocular artery Doppler could improve the performance of PE screening, especially for preterm PE. Additionally, it demonstrated that the PSV ratio outperformed MAP, UtA-PI, PlGF, or sFlt-1 in PE prediction when considered as a standalone marker [17]. In comparison to serum biomarkers. such as PlGF and sFlt-1, measuring the ophthalmic artery is considerably more affordable to patients and places less economical strain on clinical practice; moreover, the measurement of the ophthalmic artery allows for spot results and prediction of PE [36,37].

Due to a lack of clinical evidence and cost effects, the majority of the major PE guidelines do not currently advocate routine screening, including biomarker measures, for the entire population [38]. As an alternative, the guideline advises identifying high-risk individuals by different means and providing preventative care. In the context of Bulgaria, a distinctive challenge arises due to the limited number of people screened for PE, particularly in the first trimester, although it is recommended by the Bulgarian national guideline to screen everyone in the first trimester [39]. The low spread of combined first-trimester screening leaves a critical gap in the early identification of individuals at risk, potentially hindering timely interventions and preventive measures. Adding an ophthalmic artery Doppler to the regular anomaly scan in Bulgaria might be a useful and quick way to improve the screening process without having to create a separate screening protocol. This integration aligns with the existing framework of prenatal care, minimizing the additional burden on both healthcare providers and pregnant individuals [40,41]. The potential impact of this implementation extends beyond the immediate identification of high-risk individuals. It may contribute to the accumulation of valuable data specific to the Bulgarian population, facilitating ongoing research efforts to refine risk prediction models and optimize preventative strategies tailored to the local context.

### Limitations

This study’s primary limitation is the small sample size investigated, which affects the sensitivity and specificity of Doppler screening for PE in pregnancy. It is also important to note that this study was a single-center study, thus limiting a wide-spread study comparison. The population of subjects used in this study was limited to one ethnic group (Caucasians). A cost–benefit analysis was not carried out in this study; therefore, a targeted recommendation cannot be made through this investigation.

While it is worth noting that measuring the ophthalmic artery will require physicians to be trained to carry out the scan, it is also one of the simplest vessels for Doppler scanning in the field of sonography and can be easily learned [17,18,25].

## 5. Conclusions

Ophthalmic artery screening has great potential for the early detection of PE, allowing for timely intervention and management. Similar to the Doppler examination of the uterine artery screening for PE in pregnancy, the Doppler investigation of the maternal ophthalmic artery is also a simple, legitimate, and accurate method with a good predictive diagnostic value for the occurrence of PE in pregnancy. The PSV2/PSV1 ratio of the ophthalmic artery is an important biomarker in the prediction of PE, which presents a strong statistical association with PE cases, and is superior to PlGF and sFlt-1. 

Incorporating the ophthalmic artery Doppler as a screening tool for PE in Bulgaria has the potential to improve early detection, risk stratification, and overall good maternal and fetal health outcomes. While the lack of widespread endorsement of the ophthalmic artery Doppler in current guidelines may pose a challenge, the unique benefits offered by this non-invasive screening method should be given more consideration for further research to include it in the current guidelines to establish its effectiveness in Bulgaria.

## Figures and Tables

**Table 1:** Demographic and clinical profile of the study population.

Variables	Overall (*n* = 200)	High-Risk Women (*n* = 60)	Low-Risk Women (*n* = 140)	*p*
Age, years (mean ± SD)	34.1 ± 5.0	35.4 ± 4.9	33.6 ± 4.9	0.020
Age ≤ 35, % (*n*)	66.0% (132)	56.7% (34)	70.0% (98)	0.068
Age > 35, % (*n*)	34.0% (68)	43.3% (26)	30.0% (42)	
BMI, kg/m^3^ (mean ± SD)	26.7 ± 5.3	27.5 ± 5.4	26.4 ± 5.3	0.184
BMI ≤ 30 kg/m^3^ (*n*)	78.5% (157)	70% (42)	82.1% (115)	0.055
BMI > 30 kg/m^3^ (*n*)	21.5% (43)	30% (18)	17.9% (25)	
Conception method				
Spontaneous, % (*n*)	90.5% (181)	83.3% (50)	93.6% (131)	0.024
IVF, % (*n*)	9.5% (19)	16.7% (10)	6.4% (9)	
Smoking				
No, % (*n*)	88.0% (176)	95.0% (57)	85.0% (119)	0.046
Yes, % (*n*)	12.0% (24)	5.0% (3)	15.0% (21)	
GDM				
No, % (*n*)	93.5% (187)	98.3% (59)	91.4% (128)	0.070
Yes, % (*n*)	6.5% (13)	1.7% (1)	8.6% (12)	
Previous PE				
No, % (*n*)	90.0% (180)	93.3% (56)	88.6% (124)	0.304
Yes, % (*n*)	10.0% (20)	6.7% (4)	11.4% (16)	
Previous FGR				
No, % (*n*)	96.0% (192)	100% (60)	94.3% (132)	0.059
Yes, % (*n*)	4.0% (8)	0% (0)	5.7% (8)	
Women				
Multigravida, % (*n*)	11.5% (23)	6.7% (4)	13.6% (19)	0.161
Nulligravida, % (*n*)	88.5% (177)	93.3% (56)	86.4% (121)	
Acetylsalicylic acid				
No, % (*n*)	91.0% (182)	81.7% (49)	95.0% (133)	0.003
Yes, % (*n*)	9.0% (18)	18.3% (11)	5.0% (7)	
Interval last delivery ≥ 24 months (mean ± SD)	3.7 ± 3.5 (23)	3.2 ± 2.1 (4)	3.8 ± 3.8 (19)	0.743
GA at time of delivery (mean ± SD)	39.1 ± 2.0	38.6 ± 2.5	39.4 ± 1.6	0.010
GA, at which PE was diagnosed (mean ± SD)	36.2 ± 2.8	35.6 ± 3.0	37.1 ± 2.5	0.244
Did not develop PE	90.0% (180)	81.7% (49)	93.6% (131)	0.010
Developed PE	10.0% (20)	18.3% (11)	6.4% (9)	
Clinical variables				
PSV2/PSV1 ratio (mean ± SD)	0.68 ± 0.12	0.72 ± 0.09	0.65 ± 0.11	0.000
Mean Ut PI (mean ± SD)	1.23 ± 0.40	1.65 ± 0.34	1.05 ± 0.27	0.000
Ut PI MoM (mean ± SD)	1.15 ± 0.37	1.54 ± 0.32	0.99 ± 0.26	0.000
MAP (mean ± SD)	86.08 ± 7.55	91.56 ± 6.06	83.74 ± 6.90	0.000
MAP MoM (mean ± SD)	1.00 ± 0.08	1.05 ± 0.71	0.98 ± 0.08	0.000
sFLT-1 MoM (mean ± SD)	1.13 ± 0.81	1.05 ± 0.72	1.16 ± 0.85	0.382
PlGF MoM (mean ± SD)	1.12 ± 0.50	1.04 ± 0.59	1.16 ± 0.45	0.108
sFLT-1/PlGF ratio (mean ± SD)	1.38 ± 2.70	1.96 ± 4.73	1.13 ± 0.85	0.044

Preeclampsia (PE); body mass index (BMI); in vitro fertilization (IVF); gestational diabetes mellitus (GDM); preeclampsia (PE); fetal growth restriction (FGR); gestational age (GA); the ratio of the second peak systolic velocity to the first (PSV2/PSV1); uterine artery pulsatility index (UtA-PI); mean arterial pressure (MAP); multiple of the median (MoM); soluble fms-like tyrosine kinase-1 (sFlt-1); placental growth factor (PlGF).

**Table 2:** Comparison of the effectiveness of PE screening in cases who developed and who did not develop PE.

Variables (% (*n*))	Did Not Develop PE (*n* = 180)	Developed PE (*n* = 20)	*χ*2	*p*
Previous PE				
No	92.7% (162)	75.5% (18)	-	-
Yes	7.2% (18)	25.0% (2)		
Acetylsalicylic acid				
No	92.7% (167)	75.5% (15)	6.95	0.008
Yes	7.2% (13)	25.0% (5)		
Variables (mean ± SD)	Did not develop PE	Developed PE	*F*	*p*
PSV2/PSV1 ratio	0.67 ± 0.1	0.76 ± 0.1	12.07	0.001
Mean Ut PI	1.22 ± 0.4	1.29 ± 0.4	0.52	0.474
Ut PI MoM	1.15 ± 0.4	1.21 ± 0.4	0.48	0.492
MAP	85.26 ± 7.2	93.48 ± 6.3	23.74	0.000
MAP MoM	0.99 ± 0.1	1.07 ± 0.1	16.71	0.000
sFLT-1 MoM	1.16 ± 0.8	0.91 ± 0.6	1.71	0.193
PlGFMoM	1.14 ± 0.5	0.94 ± 0.4	2.94	0.088
sFLT-1/PlGF ratio	1.26 ± 1.6	2.46 ± 7.0	3.65	0.058

Preeclampsia (PE); the ratio of the second peak systolic velocity to the first (PSV2/PSV1); uterine artery pulsatility index (UtA-PI); mean arterial pressure (MAP); multiple of the median (MoM); soluble fms-like tyrosine kinase-1 (sFlt-1); placental growth factor (PlGF).

## Data Availability

Data will be provided on request.

## References

[1] Brown M.A., Lindheimer M.D., de Swiet M., Assche A.V., Moutquin J.-M. (2001). The Classification and Diagnosis of the Hypertensive Disorders of Pregnancy: Statement from the International Society for the Study of Hypertension in Pregnancy (ISSHP). Hypertens. Pregnancy.

[2] Brown M.A., Magee L.A., Kenny L.C., Karumanchi S.A., McCarthy F.P., Saito S., Hall D.R., Warren C.E., Adoyi G., Ishaku S. (2018). The Hypertensive Disorders of Pregnancy: ISSHP Classification, Diagnosis & Management Recommendations for International Practice. Pregnancy Hypertens..

[3] Chang K.-J., Seow K.-M., Chen K.-H. (2023). Preeclampsia: Recent Advances in Predicting, Preventing, and Managing the Maternal and Fetal Life-Threatening Condition. Int. J. Environ. Res. Public Health.

[4] Say L., Chou D., Gemmill A., Tunçalp Ö., Moller A.-B., Daniels J., Gülmezoglu A.M., Temmerman M., Alkema L. (2014). Global Causes of Maternal Death: A WHO Systematic Analysis. Lancet Glob. Health.

[5] Adekanmi A., Olatunji R., Obajimi M., Roberts O., Ojo T. (2015). Maternal Ophthalmic Artery Doppler Velocimetry in Pre-Eclampsia in Southwestern Nigeria. Int. J. Women’s Health.

[6] Gonser M., Vonzun L., Ochsenbein-Kölble N. (2022). Ophthalmic Artery Doppler as a Marker of Pre-eclampsia: Why Does It Work?. BJOG Int. J. Obstet. Gynaecol..

[7] Kane S.C., Brennecke S.P., da Silva Costa F. (2016). Ophthalmic Artery Doppler Analysis: A Window into the Cerebrovasculature of Women with Pre-eclampsia. Ultrasound Obstet. Gynecol..

[8] Gonser M., Vonzun L., Ochsenbein-Kölble N. (2021). Ophthalmic Artery Doppler in Prediction of Pre-eclampsia: Insights from Hemodynamic Considerations. Ultrasound Obstet. Gynecol..

[9] Mackensen F., Paulus W.E., Max R., Ness T. (2014). Ocular Changes During Pregnancy. Dtsch. Aerzteblatt Online.

[10] Sharudin S.N., Saaid R., Samsudin A., Mohamad N.F. (2020). Subfoveal Choroidal Thickness in Pre-Eclampsia. Optom. Vis. Sci..

[11] Abu Samra K. (2013). The Eye and Visual System in the Preeclampsia/Eclampsia Syndrome: What to Expect?. Saudi J. Ophthalmol..

[12] Diniz A.L.D., Paes M.M.B.M. (2022). Ophthalmic Artery Doppler in Hypertensive Pregnancies: Small Vessel, Many Possibilities. BJOG Int. J. Obstet. Gynaecol..

[13] Matias D.S., Costa R.F., Matias B.S., Cláudio Lemos Correia L. (2012). Doppler Velocimetry of the Orbital Vessels in Pregnancies Complicated by Preeclampsia. J. Clin. Ultrasound.

[14] Hata T., Hata K., Moritake K. (1997). Maternal Ophthalmic Artery Doppler Velocimetry in Normotensive Pregnancies and Pregnancies Complicated by Hypertensive Disorders. Am. J. Obstet. Gynecol..

[15] Prediction of the Risk for Preeclampsia 11 + 0 to 14 + 1 Weeks. https://fetalmedicine.org/research/assess/preeclampsia/first-trimester.

[16] Vlachopoulos C., O’Rourke M., Nichols W.W. (2011). McDonald’s Blood Flow in Arteries: Theoretical, Experimental and Clinical Principles.

[17] Sapantzoglou I., Wright A., Arozena M.G., Campos R.V., Charakida M., Nicolaides K.H. (2020). Ophthalmic Artery Doppler in Combination with Other Biomarkers in Prediction of Pre-eclampsia at 19–23 Weeks’ Gestation. Ultrasound Obstet. Gynecol..

[18] Sarno M., Wright A., Vieira N., Sapantzoglou I., Charakida M., Nicolaides K.H. (2020). Ophthalmic Artery Doppler in Prediction of Pre-eclampsia at 35–37 Weeks’ Gestation. Ultrasound Obstet. Gynecol..

[19] Selima E.R., Abar A.M., Dessouky B.A.E. (2022). Role of Ophthalmic Artery Doppler in Prediction of Preeclampsia. Egypt. J. Hosp. Med..

[20] Dimitrova V., Stratieva V. (2011). Recommendations of the Bulgarian Society of Obstetrics and Gynecology for Preeclampsia.

[21] Litwinska M., Syngelaki A., Wright A., Wright D., Nicolaides K.H. (2018). Management of Pregnancies after Combined Screening for Pre-eclampsia at 19–24 Weeks’ Gestation. Ultrasound Obstet. Gynecol..

[22] Mynard J.P., Kowalski R., Cheung M.M.H., Smolich J.J. (2016). Beyond the Aorta: Partial Transmission of Reflected Waves from Aortic Coarctation into Supra-Aortic Branches Modulates Cerebral Hemodynamics and Left Ventricular Load. Biomechan. Model. Mechanobiol..

[23] Mills C.J., Gabe I.T., Gault J.H., Mason D.T., Ross J., Braunwald E., Shillingford J.P. (1970). Pressure-Flow Relationships and Vascular Impedance in Man. Cardiovasc. Res..

[24] Erickson S.J., Hendrix L.E., Massaro B.M., Harris G.J., Lewandowski M.F., Foley W.D., Lawson T.L. (1989). Color Doppler Flow Imaging of the Normal and Abnormal Orbit. Radiology.

[25] Nicolaides K.H., Sarno M., Wright A. (2021). Ophthalmic Artery Doppler in the Prediction of Preeclampsia. Am. J. Obstet. Gynecol..

[26] Wright D., Syngelaki A., Akolekar R., Poon L.C., Nicolaides K.H. (2015). Competing Risks Model in Screening for Preeclampsia by Maternal Characteristics and Medical History. Am. J. Obstet. Gynecol..

[27] Aracil Moreno I., Rodríguez-Benitez P., Ruiz-Minaya M., Bernal Claverol M., Ortega Abad V., Hernández Martin C., Pintado Recarte P., Yllana F., Oliver-Barrecheguren C., Álvarez-Mon M. (2021). Maternal Perinatal Characteristics in Patients with Severe Preeclampsia: A Case-Control Nested Cohort Study. Int. J. Environ. Res. Public Health.

[28] Thomopoulos C., Salamalekis G., Kintis K., Andrianopoulou I., Michalopoulou H., Skalis G., Archontakis S., Argyri O., Tsioufis C., Makris T.K. (2016). Risk of Hypertensive Disorders in Pregnancy Following Assisted Reproductive Technology: Overview and Meta-analysis. J. Clin. Hypertens..

[29] Wright A., Wright D., Ispas C.A., Poon L.C., Nicolaides K.H. (2015). Mean Arterial Pressure in the Three Trimesters of Pregnancy: Effects of Maternal Characteristics and Medical History. Ultrasound Obstet. Gynecol..

[30] Tayyar A., Krithinakis K., Wright A., Wright D., Nicolaides K.H. (2016). Mean Arterial Pressure at 12, 22, 32 and 36 Weeks’ Gestation in Screening for Pre-Eclampsia. Ultrasound Obstet. Gynecol..

[31] Antwi E., Amoakoh-Coleman M., Vieira D.L., Madhavaram S., Koram K.A., Grobbee D.E., Agyepong I.A., Klipstein-Grobusch K. (2020). Systematic Review of Prediction Models for Gestational Hypertension and Preeclampsia. PLOS ONE.

[32] Papageorghiou A.T., Yu C.K.H., Bindra R., Pandis G., Nicolaides K.H. (2001). Multicenter Screening for Pre-eclampsia and Fetal Growth Restriction by Transvaginal Uterine Artery Doppler at 23 Weeks of Gestation. Ultrasound Obstet. Gynecol..

[33] Lees C., Parra M., Missfelder-Lobos H., Morgans A., Fletcher O., Nicolaides K.H. (2001). Individualized Risk Assessment for Adverse Pregnancy Outcome by Uterine Artery Doppler at 23 Weeks. Obstet. Gynecol..

[34] Stoilov B., Zaharieva-Dinkova P., Stoilova L., Uchikova E., Karaslavova E. (2023). Independent Predictors of Preeclampsia and Their Impact on the Complication in Bulgarian Study Group of Pregnant Women. Folia Medica.

[35] Matias D.S., Costa R.F., Matias B.S., Gordiano L., Correia L.C.L. (2014). Predictive Value of Ophthalmic Artery Doppler Velocimetry in Relation to Development of Pre-eclampsia. Ultrasound Obstet. Gynecol..

[36] Kumari N., Ranjan R.K., Rai N., Xalxo A.R., Toppo S.K., Ram P.N. (2023). A Correlational Study of Ophthalmic Artery Doppler Parameters and Maternal Blood Pressure in Normotensive and Pre-Eclamptic Pregnancies at a Tertiary Care Hospital. Cureus.

[37] Gao J., Shen J., Jiang Y., Zhou X., Qi H., Liu X., Liu J., Yang J., Bian X. (2014). Value of second trimester maternal serum sFlt-1, PlGF and their ratio in the prediction of preeclampsia. Zhonghua Fu Chan Ke Za Zhi.

[38] UK National Screening Committee (2022). Screening for Prediction and Prevention of Pre-Eclampsia.

[39] Stratieva V. (2016). Placental Dysfunction-Screening Model for Risk Assessment. Ph.D. Thesis.

[40] Poon L.C., Sahota D. (2019). Screening and Prevention of Preeclampsia. Matern. Med..

[41] August P., Jeyabalan A. Preeclampsia: Prevention. https://www.uptodate.com/contents/preeclampsia-prevention?sectionName=LOW-DOSE%20ASPIRIN.

